# Pygenomics: manipulating genomic intervals and data files in Python

**DOI:** 10.1093/bioinformatics/btad346

**Published:** 2023-05-25

**Authors:** Gaik Tamazian, Nikolay Cherkasov, Alexander Kanapin, Anastasia Samsonova

**Affiliations:** Institute for Translational Biomedicine, Saint Petersburg State University, St. Petersburg 199034, Russia; Institute for Translational Biomedicine, Saint Petersburg State University, St. Petersburg 199034, Russia; Institute for Translational Biomedicine, Saint Petersburg State University, St. Petersburg 199034, Russia; Institute for Translational Biomedicine, Saint Petersburg State University, St. Petersburg 199034, Russia

## Abstract

**Summary:**

We present pygenomics, a Python package for working with genomic intervals and bioinformatic data files. The package implements interval operations, provides both API and CLI, and supports reading and writing data in widely used bioinformatic formats, including BAM, BED, GFF3, and VCF. The source code of pygenomics is provided with in-source documentation and type annotations and adheres to the functional programming paradigm. These features facilitate seamless integration of pygenomics routines into scripts and pipelines. The package is implemented in pure Python using its standard library only and contains the property-based testing framework. Comparison of pygenomics with other Python bioinformatic packages with relation to features and performance is presented. The performance comparison covers operations with genomic intervals, read alignments, and genomic variants and demonstrates that pygenomics is suitable for computationally effective analysis.

**Availability and implementation:**

The source code is available at https://gitlab.com/gtamazian/pygenomics.

## 1 Introduction

A significant part of computational analyses in bioinformatics concerns transforming data between various formats and working with intervals in assembled genome sequences (further referred to as “genomic intervals”). The intervals may represent annotated genomic features such as genes, variants, and repeats. Moreover, a vast majority of the bioinformatic data formats is based on genomic intervals accompanied by additional information. For example, the SAM format (Li *et al.* 2009) specifies genomic intervals with aligned reads, while the VCF format (Danecek *et al.* 2011) stores intervals of genomic variants in a reference genome sequence with information about alternative alleles and variant calling error probabilities.

We present pygenomics—a Python package that implements both parsing bioinformatic data and operations with intervals via red-black trees (Cormen *et al.* 1990). The package is written in pure Python, requires only its standard library, and can be run on any implementation of the language, including CPython and PyPy. The source code of pygenomics follows the functional programming paradigm: objects are immutable and functions produce no side effects except for routines related to input-output or raising exceptions. To validate the package routines, both static type checking and dynamic property-based tests are used. Pygenomics supports reading and writing for multiple bioinformatic data formats, including BAM, BED, GFF3, and VCF. We compare features and performance of pygenomics with four other bioinformatic packages implemented in Python: pysam (Heger *et al.* 2009), pybedtools (Dale *et al.* 2011), cyvcf2 (Pedersen and Quinlan 2017), and PyRanges (Stovner and Sætrom 2020). The pygenomics routines can be easily incorporated into Snakemake pipelines or other bioinformatic data processing workflows.

## 2 Approach

Pygenomics is an extensively documented deployable package with added type annotations and test suites. The software offers both application programming and command-line interfaces (i.e. API and CLI). The API supports calling the package routines in third-party software, in Snakemake pipelines, or in an interactive Python shell like IPython or Jupyter (Perez and Granger 2007). The CLI enables pygenomics usage as a stand-alone executable and allows to pass arguments to the package routines via command-line options (see Supplementary Data Section S3.2).

### 2.1 Features

Operations with genomic intervals in pygenomics are based on red-black trees described in Cormen *et al.* (1990). We chose red-black trees for their simple yet effective implementation (Okasaki 1999) and for the fast construction procedure that requires fewer tree rebalancing operations as compared to other tree types (Pfaff 2004). The latter property is important for immutable objects that are reconstructed as opposed to being modified. The implemented operations include searching, intersecting, merging, subtracting, and getting complement intervals. Another structure for manipulating intervals is the nested containment list (Alekseyenko and Lee 2007), which implementation in C and Cython is used in the PyRanges package.

Unlike general numeric intervals, the genomic intervals are associated with assembled genome sequences (chromosomes, scaffolds, or contigs) and the interval start and end positions are specified by non-negative integers bounded by sizes of the assembled genome sequences. These features allow to implement associated operations more effectively and robustly as compared to general-purpose numeric interval libraries (see Supplementary Data Section S2).

Pygenomics implements reading and writing genomic intervals for multiple bioinformatic data formats, including BED, GTF, GFF3, WIG, VCF, SAM, and BAM (Bonfield *et al.* 2021). The package also provides routines for sequence input and output in the FASTA and FASTQ formats (Cock *et al.* 2010).

### 2.2 Implementation

The source code of pygenomics conforms to Python version 3.7, thus enabling its deployment with the latest versions of two widely used implementations of Python: CPython and PyPy. Compatibility with PyPy substantially increases performance of running pygenomics routines by taking full advantage of a large number of runtime optimizations. Details about the design principles and implementation of pygenomics and their comparison to other packages are given in Supplementary Data Section S1.

### 2.3 Validation

Pygenomics routines are validated through the static type checking and through the dynamic property-based testing. Since every object in the package has its type annotated, the compatibility between them in statements within the package routines is checked by the mypy static type checker. Mypy checks are exclusively based on the annotated and inferred types, i.e. without launching any routines from the package.

Contrary to the type checking, the property-based testing procedure runs functions that verify correct behavior of the package source code entities by representing a specific property for one or several routines to be checked. For example, the function that converts a record to a string must be inverse to the function that parses the record from a string. The property-based testing is implemented using the Hypothesis library (MacIver *et al.* 2019), that generates random input arguments for the testing functions, and the pytest framework, that runs the test functions, checks conditions within them, and summarizes the testing results.

## 3 Usage

We highlight some of pygenomics features by means of the three use cases: estimating coverage of genomic repeats by aligned reads, summarizing the transition/transversion ratio distribution by populations and genome regions, and measuring performance of reading and merging genomic intervals. We also compare pygenomics to pybedtools, PyRanges, pysam, and cyvcf2 in terms of running time and memory usage. Both use cases and performance comparisons are implemented as Snakemake pipelines and are publicly available in the online repository on GitLab (see Supplementary Data Sections S3–S5).

Neither pygenomics nor other packages outperform each other for all considered operations and input data sizes. We suppose that actual performance depends on multiple factors and real-world applications may require fine-tuning or optimization which may be easier to perform in pygenomics due to its design principles and pure Python implementation.

## 4 Conclusion

Pygenomics can be easily integrated into Snakemake pipelines using either API or CLI and facilitates generation of consistent results. The pure Python implementation of the package enables deployment of pygenomics-based pipelines on any system with the Python environment installed. Using pure Python code may reduce performance of pygenomics compared to packages that rely on external compiled libraries, but we assume that benefits of easy deployment outweigh the possible performance loss. The static type checking and property-based testing frameworks embedded in the package provide a reliable and convenient way to ensure integrity and portability of the package routines. The type annotations can be used by a developer to validate usage of pygenomics routines in their own source code. The consistent API, the functional programming paradigm, and stream-based input and output implemented in pygenomics, makes it a robust and reliable software solution for development of bioinformatic tools and for delivery of reproducible results.

## Supplementary Material

btad346_Supplementary_Data

## Data Availability

The source code of pygenomics is publicly available at the following GitLab repository: https://gitlab.com/gtamazian/pygenomics. Other data related to this article are listed in Supplementary Data Section S3.1.
