# Supplementary material for: Pygenomics: manipulating genomic intervals and data files in Python
Source: Bioinformatics. 2023 May 25;39(6):btad346. doi: 10.1093/bioinformatics/btad346 (PMC10246576; doi:10.1093/bioinformatics/btad346)
Supplement: btad346_Supplementary_Data [file btad346_supplementary_data.pdf]

Supplementary Data for "Pygenomics: manipulating genomic intervals and data files in Python"

Gaik Tamazian[1], Nikolay Cherkasov[1], Alexander Kanapin[1], and Anastasia Samsonova*[1]

[1]Institute for Translational Biomedicine, Saint Petersburg State University, St. Petersburg, 199034, Russia

May 24, 2023

This supplementary text presents *pygenomics* features and compares the package with other bioinformatic toolkits implemented in Python, namely:

- *pysam* [1]
- *pybedtools* [2]
- *cyvcf2* [3]
- *PyRanges* [4].

1 Package features

In this section we present (where possible) head-to-head feature comparison between *pygenomics* and the aforementioned packages.

1.1 Data formats

Pygenomics supports reading and writing data in multiple bioinformatic formats in accordance with their specifications. Some output formats, however, are software-specific; therefore, routines related to these formats are implemented in the separate extension package *pygenomics-ext* (see Subsection 3.1). The formats supported by *pygenomics* and their implementation statuses in other Python packages are listed in Table 1.

1.2 Implementation specifics

Pure Python implementation. *Pygenomics* is implemented in pure Python using routines from the Python standard library only. Importantly, the clean Python code in the absence of dependencies from third-party libraries or compilers facilitates easy deployment and maintenance of *pygenomics* and also enables the performance boost when using *PyPy* [5]. Unlike *pygenomics*, the other packages considered here include code fragments in another programming languages or require external libraries:

*a.samsonova@spbu.ru

Table 1: Data formats supported by the considered packages.

	pysam	pybedtools	cyvcf2	PyRanges	pygenomics
BAM	+	+		+	+
BED	+	+		+	+
FASTA	+	+		+	+
FASTQ	+				+
GFF3	+	+		+	+
GTF	+	+		+	+
SAM	+	+			+
VCF	+	+	+		+
WIG					+

- *pysam* requires the *HTSlib* library [6] for parsing files;
- *pybedtools* calls the external program, *BEDTools* [7] for operations with genomic intervals;
- *cyvcf2* is implemented in *Cython* [8] and uses routines from *HTSlib*;
- *PyRanges* is based on the *pandas* library [9] and, consequently, depends on the *NumPy* library [10].

Functional programming paradigm. *Pygenomics* routines are designed in accordance with the functional programming paradigm: objects are immutable and functions produce no side effects (i.e., *pure* functions) except for routines raising exceptions or related to input-output. For that purpose, we utilized means for functional programming provided by Python: first-class and higher-order functions, immutable tuples, enumerations, and iterators.

Immutability guarantees that *pygenomics'* objects will not be able to violate invariants of their classes after the objects are created. Immutability also provides thread safety, and thus facilitates parallel programming.

Due to the absence of side effects, combining pure functions results in a pure function as well. As pure functions return a result of their work, the returned object can be passed on to another function, thus enabling the function call chaining. Besides combining and chaining, pure functions in *pygenomics* can be optimized by replacing their Python implementations with calls to external libraries implemented in other programming languages (e.g., C or Fortran).

Other packages discussed here use regular Python classes with methods that allow in-place modification of their objects. In particular, *PyRanges* objects are based on *pandas'* data frames, which methods implement changing their contents by destructing their previous state.

Property-based testing framework. In addition to source code validation, a testing framework can be used for both deploying a package and its integrity maintenance. When deploying a package, an administrator launches tests to ensure that the environment is suitable for the package. Whenever a developer introduces changes to the code tests could be run to check that proposed modifications to the source code do not break other components of the package.

Due to *pygenomics'* adherence to the functional programming paradigm, the package is provided with the property-based testing framework as implemented in the *Hypothesis* library [11]. *Hypothesis*

allows to create test cases by specifying properties of routines being tested and by passing randomly generated data to the routines to check whether the properties hold. Randomly generated testing framework enables easy development and deployment of *pygenomics* since a developer does not need to design the tests manually. Moreover, edge cases of input parameters to the tested routines are also automatically covered.

Pysam, *pybedtools* and *cyvcf2* use test cases based on the static testing datasets provided with the packages. *PyRanges* includes the property-based testing framework that just as *pygenomics* uses *Hypothesis*. However, its implementation is less efficient because *PyRanges* does not follow the functional programming paradigm and contains complex classes which random sampling requires a lot of computations and time.

Type annotations and static type checking. Although Python is a dynamically typed language, it allows a developer to annotate object types. In addition to source code validation, the type annotations facilitate code development by providing hints about package entities and by enabling detection of errors caused by passing parameters of incorrect types to the package routines.

Every entity in the source code of *pygenomics* has its type explicitly annotated. Consistencies between the entity types are checked by the *mypy* static type checker [12]. This procedure does not require any calls of *pygenomics* routines. The type annotations are also installed when *pygenomics* is deployed, thus allowing a user to refer to types of *pygenomics'* routines when developing their own code.

The other packages do not have type annotations.

Both API and CLI provided. *Pygenomics* provides both the application programming interface (API) and the command-line interface (CLI) to its routines. The API gives access to all routines of the package and the CLI enables to run the package routines as ordinary programs outside a development environment. This is a significant advantage as packages mentioned above do not provide CLI.

Consistent API. The API of *pygenomics* is developed with the intention to keep it consistent and easy to comprehend. Thus, the following principles are observed to maintain the API integrity:

1. Routines for parsing data are organized into modules or subpackages (e.g., subpackage `pygenomics.vcf` contains routines related to reading various parts of a VCF file).

2. Exception classes are organized into a downward hierarchy that starts from the base class for exceptions in the package (`pygenomics.error.Error`). Base exception classes are also present in modules and subpackages of *pygenomics* (e.g., `pygenomics.vcf.error.Error`). Classes for specific exceptions inherit from their base classes to allow catching the exceptions at any level of the package (e.g., exceptions for a specific subpackage or all exceptions possibly generated by *pygenomics*).

3. Routines shared between modules and subpackages are organized into a downward hierarchy of modules which are named `common` (e.g., `pygenomics.common`). Similar to the exception classes, the modules for common routines import only higher-level modules to avoid circular dependencies (e.g., `pygenomics.vcf.meta.common` imports `pygenomics.common`).

4. A number of idioms are used in the package routines:

   (a) Classes that represent bioinformatic data file items are called `Record` (the examples are `pygenomics.bed.Record` and `pygenomics.gff3.Record`).

(b) Names of classes representing module-level errors end with `Error`. Such classes might be accompanied by enumeration-based classes which names end with `ErrorType` and that allows for the specification of the error type (e.g., `pygenomics.fastq.ReadingError` and `pygenomics.fastq.ReadingErrorType`).

(c) Methods that implement conversion of a line or a list of strings into class objects are called `of_string()` or `of_list()`, respectively. These methods return the class object, if the conversion was successful or `None` otherwise.

Pysam and *cyvcf2* wrap code from *HTSlib* and their APIs depend on the *HTSlib* API, which is implemented in C. The API of *pybedtools* is designed to pass data to programs from *BEDTools* and parse their output. The API of *PyRanges* seems to be influenced by *pandas'* API and is based on the data frame objects.

Structured CLI. The CLI of *pygenomics* organizes its commands into categories by the kind of data the commands are applied to (e.g., file formats or operation types). Each command is provided with a help message that describes its command-line arguments. The CLI commands are described in Subsection 3.2.

The other packages we consider here do not provide CLI.

Source code design. *Pygenomics* is developed following the best practices for Python programming: arranging routines in subpackages and modules, annotating types, documenting entities in their source code, and, finally, assessing the code complexity to avoid bloated classes or functions. The development framework established for *pygenomics* is described in Subsection 1.3.

The source code of *Pysam, cyvcf2* and *pybedtools* is affected by their dependency on external tools or libraries (that is, *BEDTools* and *HTSlib*). *PyRanges* is based on the *pandas* package, and thus its source code adheres to design principles used to develop *pandas* and *NumPy*. These design principles are aimed at maximizing performance of operations with large data sets (series and data frames in *pandas* and multidimensional arrays in *NumPy*) by allowing modification of existing instances of the data objects. Such in-place modifications increase performance but also introduce side effects that may have undesirable consequences. For example, *pandas* (version 1.5.1) is known not to be completely thread safe [13]. Furthermore, *NumPy* introduces its own development roadmap and a series of enhancement proposals called NEPs (NumPy Enhancement Proposals) [14] in addition to the roadmap and proposals for Python [15]. Tracking NEPs might be excessive for a user or developer of bioinformatic software that does not explicitly involve operating with multidimensional arrays.

Stream-based input and output. *Pygenomics* routines use streams for specifying sources and destinations of input and output (I/O). Using streams provides a user with a flexible way to process I/O errors outside the routines. The streams can be connected to files, network resources or located in memory.

The other packages mentioned here use file names for specifying I/O sources.

Flexible parsing of bioinformatic data. Methods that parse bioinformatic data from a line or a series of strings in *pygenomics'* classes return `None` if the parsing fails. This behavior allows a user to specify their own strategy for dealing with data that cannot be parsed e.g., to terminate the program, to collect the parsing failures for further processing, to try correcting the failures, or to ignore them completely.

A user can also create an intermediate function for processing non-standard input data (e.g., spaces in lines from a non-standard BED file can be replaced with tab characters before passing these lines to the function that parses BED records). This approach is far more efficient than implementation of

handling of multiple variants of a data format (e.g., allowing several column separators). It adapts to irregularities in input data and imposes no computational burden by processing redundant variants of a data format.

Pysam, *cyvcf2* and *pybedtools* raise exceptions if incorrect input data is parsed. *PyRanges* allows variants of input data formats, but does not give an opportunity to specify custom variants without introducing changes to the source code of the package.

Classes allow easy querying. *Pygenomics* classes are designed to enable easy exploration of their content during a read-eval-print loop (REPL) in the Python interpreter. Each class has its representation (that is returned by function `repr()`) defined in the human-readable format and type annotations facilitate perusal of the source code.

Pysam, *cyvcf2* and *pybedtools* wrap data structures from other libraries or programs that cannot be conveniently viewed in the REPL. *PyRanges* provides viewing its objects in the REPL in a manner similar to *pandas*.

1.3 Development framework

Pygenomics has been developed using a number of programming tools that facilitate writing clear code and maintaining the package integrity:

- *Black* [16] keeps the source code of *pygenomics* formatted in the same style and in accordance with widely used conventions for Python source code formatting.
- *isort* [17] manages the hierarchy of the `import` statements in the source code.
- *pylint* [18] detects incorrect or improper code and assists with refactoring the codebase of *pygenomics* (e.g., by reporting duplicated fragments of code).
- *mypy* [12] checks type annotations and reports errors caused by inconsistent types.
- *radon* [19] assesses source code complexity for the package entities (classes, methods, and functions) and estimates maintainability of the package codebase based on cyclomatic complexity [20].
- *flake8* [21] performs extra static code checks.
- *pytest* [22] provides the source code testing framework.
- *Hypothesis* [11] implements property-based testing that was introduced in the Haskell library *Quickcheck* [23].
- *Sphinx* [24] extracts in-source documentation of the package entities and converts it to a number of output formats, including HTML and PDF.
- *tox* [25] organizes running tests and static code checks in the way that enables convenient integration with external continuous integration (CI) tools.
- *Poetry* [26] manages packaging and dependencies of *pygenomics* in a portable way. Although *pygenomics* routines do not depend on third-party libraries, its development framework does and *Poetry* guarantees that there are no version conflicts between the development framework packages and their dependencies. *Poetry* also can be used for managing a virtual environment for developing and testing *pygenomics*.

The presented development framework and the package properties listed in Subsection 1.2 facilitate continuous integration and continuous development (CI/CD) of *pygenomics*, as well as its incorporation into production-grade bioinformatic packages and pipelines.

2 Comparison with general-purpose numeric interval libraries

A number of general-purpose libraries that implement numeric interval operations have been developed. These libraries include *Boost Interval Arithmetic Library* [27] and *intervaltree* [28].

Boost Interval Arithmetic Library is a template-based C++ library that implements manipulating mathematical intervals. The library is indented for various applications, including computer graphics and interval calculation for imprecise data. *Intervaltree* is a Python package that provides a mutable self-balancing interval tree originally designed for text and time intervals. Both libraries allow a developer to specify the type of interval bounds, which might be a floating-point number type.

Unlike general numeric intervals, genomic intervals are associated with assembled genome sequences (chromosomes, scaffolds, or contigs) and the interval start and end positions are specified by non-negative integers bounded by sizes of the sequences. *Pygenomics* uses these features of genomic intervals for effective and robust implementation of the associated operations. For example, an attempt to create a BED record range with a negative start position will raise an exception:

```
>>> import pygenomics.bed
>>> pygenomics.bed.Range(-1, 1)
pygenomics.bed.BedError: BedErrorType.INCORRECT_RANGE
```

Boundaries for end positions of genomic intervals vary according to a genome assembly or might be undefined. A developer can implement extra routines for restricting the interval end coordinates. For example, the *pygenomics interval complement* command from *pygenomics'* CLI allows a user to specify genome sequence sizes: if the sizes are specified, then intervals at the beginning and end of the sequences are included in the tool output. Otherwise, only intervals located between the given ones are reported.

Boost Interval Arithmetic Library and *intervaltree* do not provide routines for working with genomic intervals, although such routines can be implemented using these libraries. The example is *intervaltree-bio* [29], that is a Python package based on *intervaltree*. However, *intervaltree-bio* is designed primarily for working with UCSC Genome Browser annotation records and does not support multiple bioinformatic data formats.

3 Using the package

3.1 Extra repositories

In addition to the main GitLab repository of *pygenomics*, we present three repositories with routines related to the package.

pygenomics-ext provides routines for processing data in non-standard or software-specific formats (https://gitlab.com/gtamazian/pygenomics-ext).

pygenomics-examples stores a collection of stand-alone scripts that utilize routines of *pygenomics* and can be run in a command line (https://gitlab.com/gtamazian/pygenomics-examples).

pygenomics-paper contains Snakemake pipelines that accompany this paper as presented further in Sections 4 and 5 (https://gitlab.com/gtamazian/pygenomics-paper).

3.2 Command-line interface

Pygenomics provides the command-line interface (CLI) to its routines related to genomic intervals or processing FASTA files. Having the package installed, the CLI can be invoked by calling script

Table 2: Command-line interface commands of *pygenomics*.

Group	Command	Description
`fasta`	`oneseq`	Extract a single specified sequence.
	`revcomp`	Produce reverse complements of nucleotide sequences.
	`size`	Print names and lengths of sequences.
	`unmask`	Remove soft masking from sequences.
`interval`	`complement`	Get complement genomic intervals.
	`find`	Find genomic intervals that overlap with the target.
	`find_all`	Print query genomic intervals that overlap with the target; the printed intervals are followed by shared intervals between the query and the target.
	`intersect`	Intersect genomic intervals from two or more files.
	`merge`	Merge genomic intervals from one or several files.
	`subtract`	Subtract genomic intervals; several files of intervals to be subtracted can be specified.

`pygenomics` or by passing the module option to Python in the following way: `python3 -m pygenomics`. The second method allows to specify the Python interpreter used for running *pygenomics* (e.g., one may use *PyPy* instead of the default Python interpreter installed in the environment: `pypy3 -m pygenomics`) or to run the CLI routines from the source code tree without having the package installed:

```
git clone https://gitlab.com/gtamazian/pygenomics.git
cd pygenomics/src
python3 -m pygenomics
```

Commands of the CLI are arranged by the type of data they process: genomic intervals or FASTA files. The list of the CLI commands is given in Table 2. Commands in the `interval` group support input files in the BED, GFF3, GTF and VCF formats. The files of different formats can be combined in a single command; conversions between interval formats (e.g., zero-based half-open for the BED format and one-based closed for the GFF3 format) are performed automatically.

4 Examples

This section presents three pipelines that demonstrate *pygenomics* usage in bioinformatics data analysis. The pipelines are implemented in *Snakemake* [30, 31] and stored in a separate repository (see Subsection 3.1). In addition to Python scripts that use *pygenomics*, the pipelines include scripts in R [32] for visualizing data generated by the Python scripts. The R scripts use routines from *ggplot2* [33] and *tidyverse* [34] packages.

4.1 Repeat coverage by aligned reads

The pipeline calculates coverage of genomic repeats in the human genome by aligned reads. For each repeat, the base-level coverage profile is produced and the mean coverage is derived. The input data includes the repeat annotation by *RepeatMasker* [35] and read alignments to chromosome 20 for individual HG00448 sequenced in the framework of the 1000 Genomes project [36]. The mean coverage values are summarized for each repeat class from the RepeatMasker annotation and the per-class coverage distribution is shown by a joint box plot in Figure 1.

4.2 Distribution of transition to transversion ratio

The pipeline computes a Ti/Tv ratio across genomic regions of different types (namely, exons, introns, long non-coding RNAs, and intergenic regions). The input data for the pipeline are a) biallelic SNPs detected on chromosome 20 from the 1000 Genomes project [37], and b) gene annotation from the NCBI Eukaryotic Genome Annotation Pipeline [38]. For every genotyped individual, heterozygous SNPs are counted for every type of genomic regions and the transition to transversion ratios (Ti/Tv) are summarized by ancestry of the individuals as presented in [39]. The box plots that visualize the Ti/Tv distribution by the genomic regions and the ancestry are shown in Figure 2.

4.3 Comparing performance of CPython and PyPy

We assessed *pygenomics* performance using two Python implementations: *CPython* (version 3.10.1) and *PyPy* (version 7.3.9). Average time required to read RepeatMasker entries for the human genome and to merge the repeat intervals was tracked for nine sets of various sizes sampled from the RepeatMasker annotation file. The benchmark was performed on the Dell™ Precision™ 3440 SFF workstation equipped with the Intel® Core™ i7-10700 CPU and 128 GB of RAM. The running time was measured using the *hyperfine* program [40] and the results are shown in Figure 3. Error bars in the figure designate two-sided 95% confidence intervals for the mean running time based on the normal distribution.

For each input set, there was one warm-up run to reduce delay effects caused by reading from a disk and ten subsequent runs to record running time. Figure 3 shows that *PyPy* accelerates running routines of *pygenomics* compared to *CPython* and the difference in performance grows with the increase of the input data size.

5 Comparative performance analysis

We compared performance of *pygenomics* with the aforementioned Python packages: *cyvcf2*, *pybedtools*, *pysam*, and *PyRanges*. Running time and maximum memory usage were recorded. All runs were performed without using parallelization capabilities of the packages. Each run was repeated thirty times on the Dell™ OptiPlex™ 5070 personal computer equipped with the Intel® Core™ i5-9600 CPU and 16 GB of RAM and two-sided 95% confidence intervals for the mean values of the time and memory usage were obtained. In the following figures, the mean values and the confidence intervals are shown by points and error bars, respectively. Ten samples of input data with equally ranged sizes were prepared for each run. Versions of the packages and their dependencies that were used in the comparison analysis are given in Table 3.

5.1 Genomic intervals

We compared performance of genomic interval operations between *pygenomics*, *pybedtools*, and *PyRanges*. The operations included intersecting, subtracting, and detecting overlapping intervals. The

Table 3: Versions of software packages and libraries involved in the comparative performance analysis. The *BEDTools* package is required for Python package *pybedtools*. The *HTSlib* library is required for Python packages *pysam* and *cyvcf2*. The *PyRanges* package requires Python packages *NumPy* and *pandas*.

Compared packages		Dependencies	
Software	Version	Software	Version
pygenomics	0.1.1	BEDTools	2.30.0
pybedtools	0.9.0	HTSlib	1.13
PyRanges	0.0.120	NumPy	1.21.5
pysam	0.20.0	pandas	1.3.5
cyvcf2	0.30.18	CPython	3.10.6
		PyPy	7.3.11

BEDTools toolkit was also installed for running *pybedtools*.

Intervals of genomic repeats annotated by *RepeatMasker* in the human genome were used as a query set. Two target sets were used: intervals of protein-coding genes from the NCBI human genome annotation and intervals of the gene exons. The gene set served as a medium-size collection of contiguous genomic intervals, while the exon set represented numerous but short intervals.

Performance measurement results for the gene and exon query sets are shown in Figures 4 and 5. The results show different rankings based on the query set, the interval operation, and the measured performance value (time or memory). Unlike *pygenomics* and *PyRanges*, the *pybedtools* package created temporary files for passing data to *BEDTools* and thus performed extra I/O operations.

5.2 Read alignments

Performance of routines for reading a BAM file was compared between *pygenomics* and *pysam*. The benchmarking script iterated read alignments in a BAM file and counted properly aligned reads. The BAM file of low-coverage sequencing reads from the 1000 Genomes project was used as input data.

Pysam outperformed *pygenomics* in both running time and maximum memory usage, as shown in Figure 6. We assume that the high performance of *pysam* could be explained by its being a Python wrapper for routines from the *HTSlib* library, that was implemented in C.

5.3 Genomic variants

Performance of routines for reading a VCF file was compared between *pygenomics*, *pysam*, and *cyvcf2*. The benchmarking script iterated variant records in a VCF file, filtered biallelic SNPs, and printed them in the HGVS notation [41]. The VCF file of genomic variants for multiple individuals from the 1000 Genomes project was used as input data.

As shown in Figure 7, *pygenomics* outperformed other packages in running time but used the greatest amount of memory.

References

[1] Andreas Heger, Kevin Jacobs, et al. *Pysam: HTSlib interface for Python*. 2009–2022. URL: https://github.com/pysam-developers/pysam (visited on 05/08/2022).

[2] Ryan K. Dale, Brent S. Pedersen, and Aaron R. Quinlan. “Pybedtools: a flexible Python library for manipulating genomic datasets and annotations.” In: *Bioinformatics* 27.24 (2011), pp. 3423–3424. DOI: 10.1093/bioinformatics/btr539.

[3] Brent S Pedersen and Aaron R Quinlan. “cyvcf2: fast, flexible variant analysis with Python.” In: *Bioinformatics* 33.12 (2017), pp. 1867–1869. DOI: 10.1093/bioinformatics/btx057.

[4] Endre Bakken Stovner and Pål Sætrom. “PyRanges: efficient comparison of genomic intervals in Python.” In: *Bioinformatics* 36.3 (2019), pp. 918–919. DOI: 10.1093/bioinformatics/btz615.

[5] The PyPy Team. *PyPy: A fast, compliant alternative implementation of Python*. 2003–2022. URL: https://www.pypy.org (visited on 05/11/2022).

[6] James K Bonfield et al. “HTSlib: C library for reading/writing high-through-put sequencing data.” In: *GigaScience* 10.2 (2021). DOI: 10.1093/gigascience/giab007.

[7] Aaron R. Quinlan and Ira M. Hall. “BEDTools: a flexible suite of utilities for comparing genomic features.” In: *Bioinformatics* 26.6 (2010), pp. 841–842. DOI: 10.1093/bioinformatics/btq033.

[8] Stefan Behnel et al. “Cython: The best of both worlds.” In: *Computing in Science & Engineering* 13.02 (2011), pp. 31–39. DOI: 10.1109/MCSE.2010.118.

[9] Wes McKinney. “Data structures for statistical computing in Python.” In: *Proceedings of the 9th Python in Science Conference*. Ed. by Stéfan van der Walt and Jarrod Millman. 2010, pp. 56–61. DOI: 10.25080/Majora-92bf1922-00a.

[10] Charles R. Harris et al. “Array programming with NumPy.” In: *Nature* 585.7825 (2020), pp. 357–362. DOI: 10.1038/s41586-020-2649-2.

[11] David MacIver, Zac Hatfield-Dodds, et al. “Hypothesis: A new approach to property-based testing.” In: *Journal of Open Source Software* 4.43 (2019), p. 1891. DOI: 10.21105/joss.01891.

[12] Jukka Lehtosalo et al. *Mypy: An optional static type checker for Python*. 2014–2022. URL: http://mypy-lang.org (visited on 05/11/2022).

[13] The pandas development team. *Frequently Asked Questions (FAQ)—pandas 1.5.1 documentation: Thread-safety*. 2022. URL: https://pandas.pydata.org/docs/user_guide/gotchas.html#thread-safety (visited on 11/12/2022).

[14] Jarrod Millman. *NEP 0—Purpose and process*. Dec. 11, 2017. URL: https://numpy.org/neps/nep-0000.html (visited on 11/12/2022).

[15] Barry Warsaw et al. *PEP 1—PEP purpose and guidelines*. June 13, 2022. URL: https://peps.python.org/pep-0001/ (visited on 11/12/2022).

[16] Łukasz Langa et al. *Black: The uncompromised code formatter*. 2018–2022. URL: https://black.readthedocs.io (visited on 05/11/2022).

[17] Timothy Crosley. *isort your imports, so you don't have to*. 2014–2021. URL: https://pycqa.github.io/isort (visited on 10/28/2022).

[18] Python Code Quality Authority. *Pylint: A Python static code analysis tool*. 2003–2022. URL: http://pylint.pycqa.org (visited on 05/11/2022).

[19] Michele Lacchia. *Radon: A Python tool that computes various metrics from the source code*. 2012–2022. URL: https://radon.readthedocs.io (visited on 05/11/2022).

[20] Thomas J. McCabe. “A complexity measure.” In: *IEEE Transactions on Software Engineering* SE-2.4 (1976), pp. 308–320. DOI: 10.1109/TSE.1976.233837.

[21] Tarek Ziadé et al. *flake8: The modular source code checker*. 2010–2022. URL: https://flake8.pycqa.org (visited on 10/29/2022).

[22] Holger Krekel et al. *Pytest: Helps you write better programs*. 2004–2022. URL: https://docs.pytest.org (visited on 05/11/2022).

[23] Koen Claessen and John Hughes. "QuickCheck: a lightweight tool for random testing of Haskell programs." In: *Proceedings of the Fifth ACM SIGPLAN International Conference on Functional Programming*. ICFP '00. New York, NY, USA: Association for Computing Machinery, 2000, pp. 268–279. DOI: 10.1145/351240.351266.

[24] Georg Brandl and the Sphinx team. *Sphinx: Python documentation generator*. 2007–2022. URL: https://www.sphinx-doc.org (visited on 05/11/2022).

[25] Bernát Gábor, Holger Krekel, Oliver Bestwalter, et al. *tox: Command line driven CI frontend and development task automation tool*. 2011–2022. URL: https://tox.wiki (visited on 10/28/2022).

[26] Sébastien Eustace et al. *Poetry: Python packaging and dependency management made easy*. 2018–2022. URL: https://python-poetry.org (visited on 10/29/2022).

[27] Guillaume Melquiond, Sylvain Pion, and Hervé Brönnimann. *Boost Interval Arithmetic Library*. Dec. 25, 2006. URL: https://www.boost.org/doc/libs/1_81_0/libs/numeric/interval/doc/interval.htm (visited on 01/06/2023).

[28] Chaim Leib Halbert and Konstantin Tretyakov. *intervaltree: a mutable, self-balancing interval tree for Python 2 and 3*. 2013–2020. URL: https://github.com/chaimleib/intervaltree (visited on 01/06/2023).

[29] Konstantin Tretyakov. *intervaltree-bio: interval tree convenience classes for genomic data*. 2015. URL: https://github.com/konstantint/intervaltree-bio (visited on 01/06/2023).

[30] Johannes Köster and Sven Rahmann. "Snakemake—a scalable bioinformatics workflow engine." In: *Bioinformatics* 28.19 (Oct. 2012), pp. 2520–2522. DOI: 10.1093/bioinformatics/bts480.

[31] Felix Mölder et al. "Sustainable data analysis with Snakemake [version 2; peer review: 2 approved]." In: *F1000Research* 10.33 (2021). DOI: 10.12688/f1000research.29032.2.

[32] R Core Team. *R: A language and environment for statistical computing*. R Foundation for Statistical Computing. Vienna, Austria, 2018. URL: https://www.R-project.org/.

[33] Hadley Wickham. *ggplot2: Elegant graphics for data analysis*. Springer-Verlag New York, 2016. ISBN: 978-3-319-24277-4. URL: https://ggplot2.tidyverse.org.

[34] Hadley Wickham et al. "Welcome to the tidyverse." In: *Journal of Open Source Software* 4.43 (2019), p. 1686. DOI: 10.21105/joss.01686.

[35] Arian F A Smit, Robert Hubley, and Philip Green. *RepeatMasker Open-4.0*. 2013–2015. URL: http://www.repeatmasker.org (visited on 06/21/2022).

[36] The 1000 Genomes Project Consortium. "An integrated map of genetic variation from 1,092 human genomes." In: *Nature* 491.7422 (2012), pp. 56–65. DOI: 10.1038/nature11632.

[37] Ernesto Lowy-Gallego et al. "Variant calling on the GRCh38 assembly with the data from phase three of the 1000 Genomes Project [version 2; peer review: 2 approved]." In: *Wellcome Open Research* 4.50 (2019). DOI: 10.12688/wellcomeopenres.15126.2.

[38] NCBI. *NCBI Homo sapiens Annotation Release 110*. 2022. URL: https://www.ncbi.nlm.nih.gov/genome/annotation_euk/Homo_sapiens/110/ (visited on 06/21/2022).

[39] Jing Wang et al. "Genome measures used for quality control are dependent on gene function and ancestry." In: *Bioinformatics* 31.3 (2015), pp. 318–323.

[40] David Peter et al. *hyperfine: A command-line benchmarking tool*. 2018–2022. URL: https://github.com/sharkdp/hyperfine (visited on 06/21/2022).

[41] Johan T. den Dunnen et al. “HGVS recommendations for the description of sequence variants: 2016 update.” In: *Human Mutation* 37.6 (2016), pp. 564–569. DOI: https://doi.org/10.1002/humu.22981.

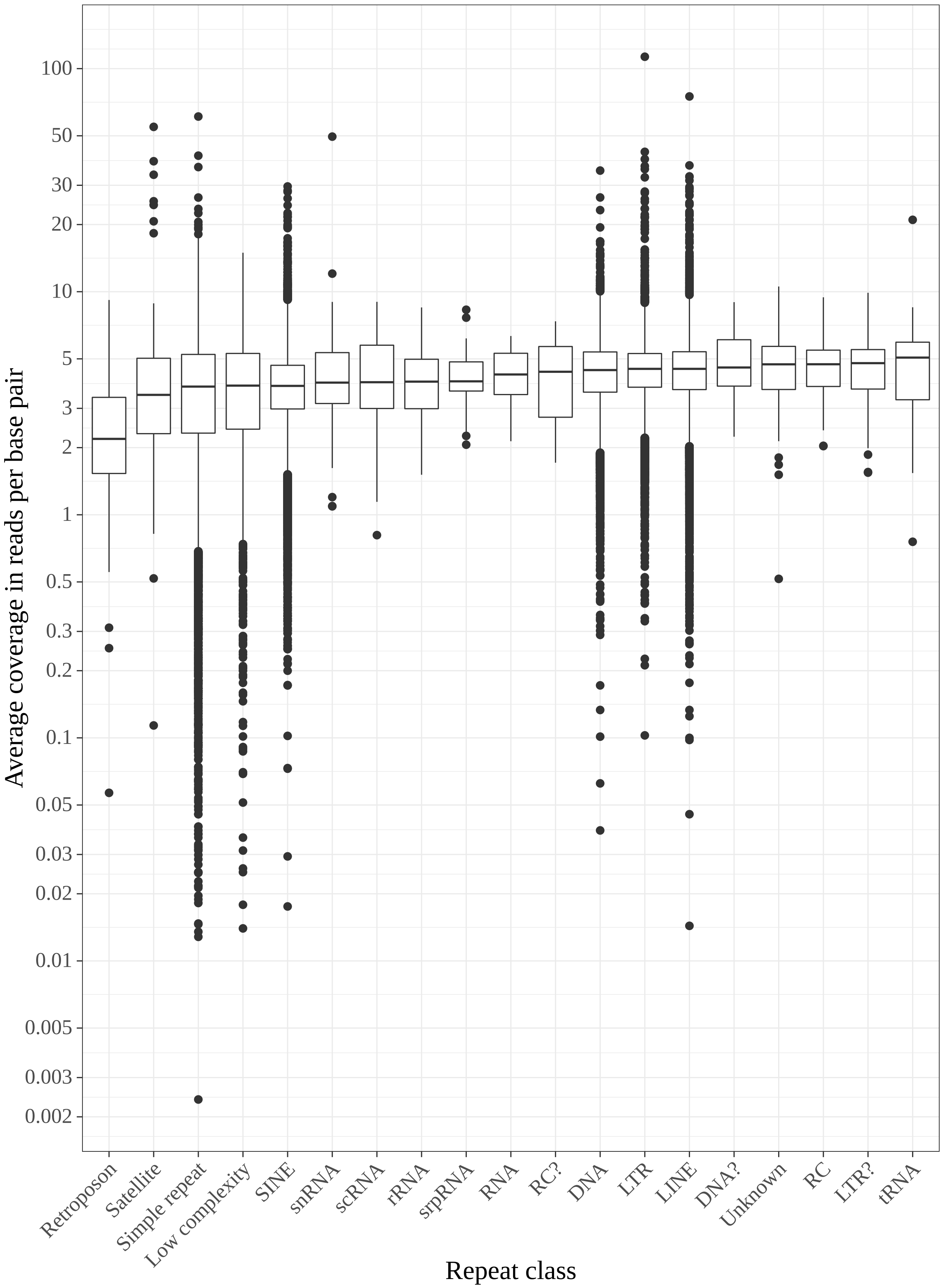

Figure 1: Distribution of mean coverage by aligned reads for genomic repeats of various classes.

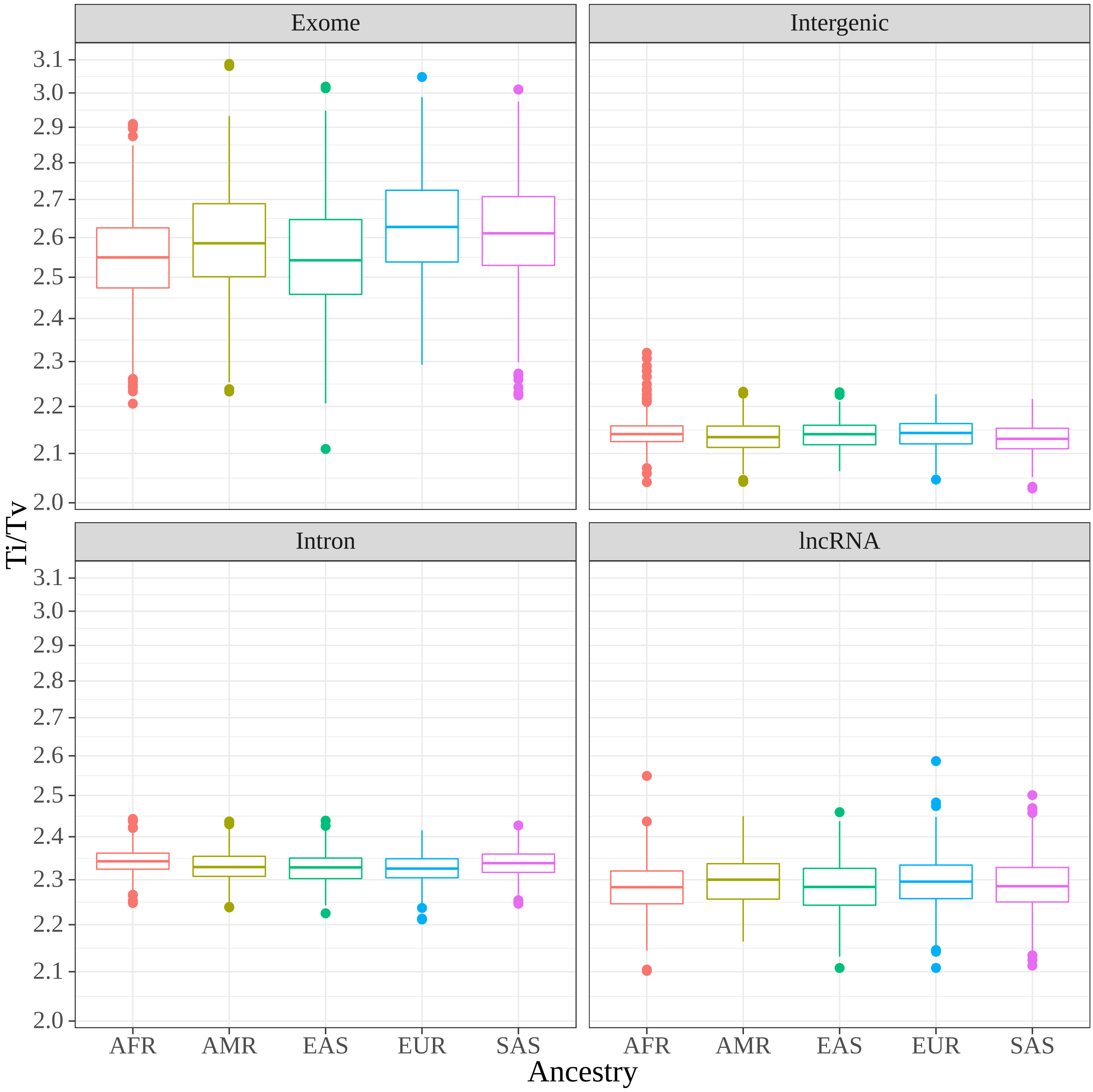

Figure 2: Distribution of transition to transversion ratios (Ti/Tv) by genomic regions and ancestry (AFR — African, AMR — American, EAS — East Asian, EUR — European, SAS — South Asian).

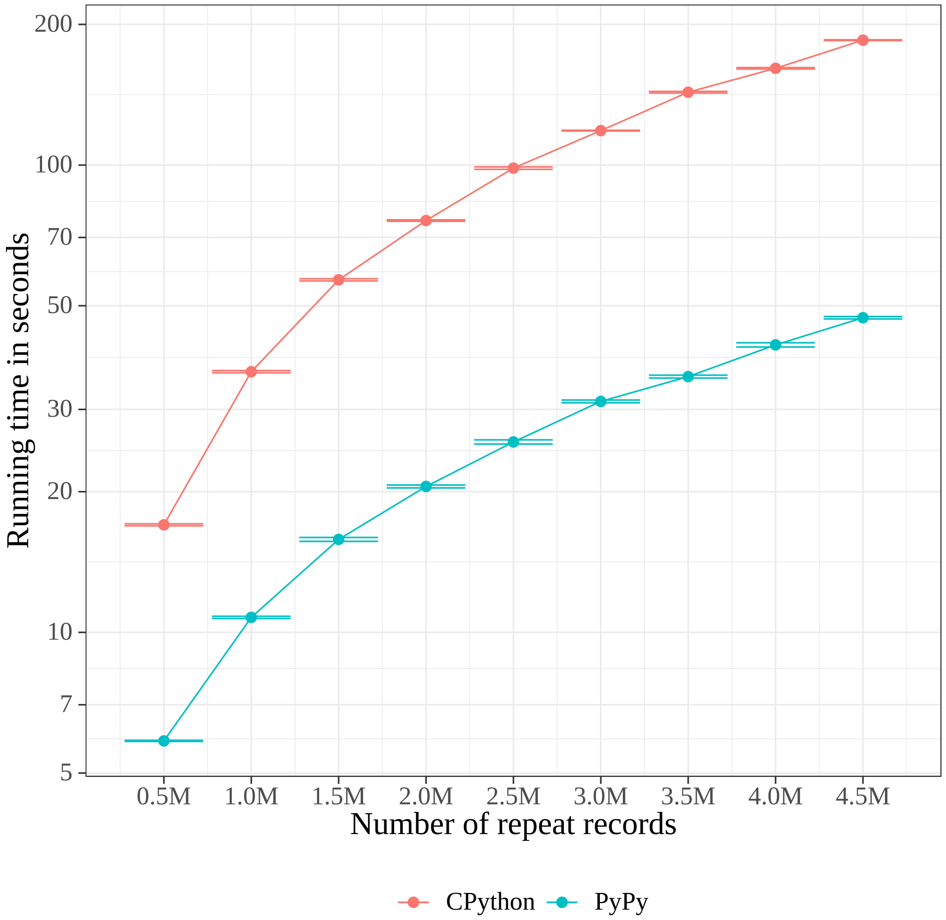

Figure 3: Running time for reading and merging of genomic intervals corresponding to human genome repeats annotated by *RepeatMasker*. Points and error bars show two-sided 95% confidence intervals for the mean values.

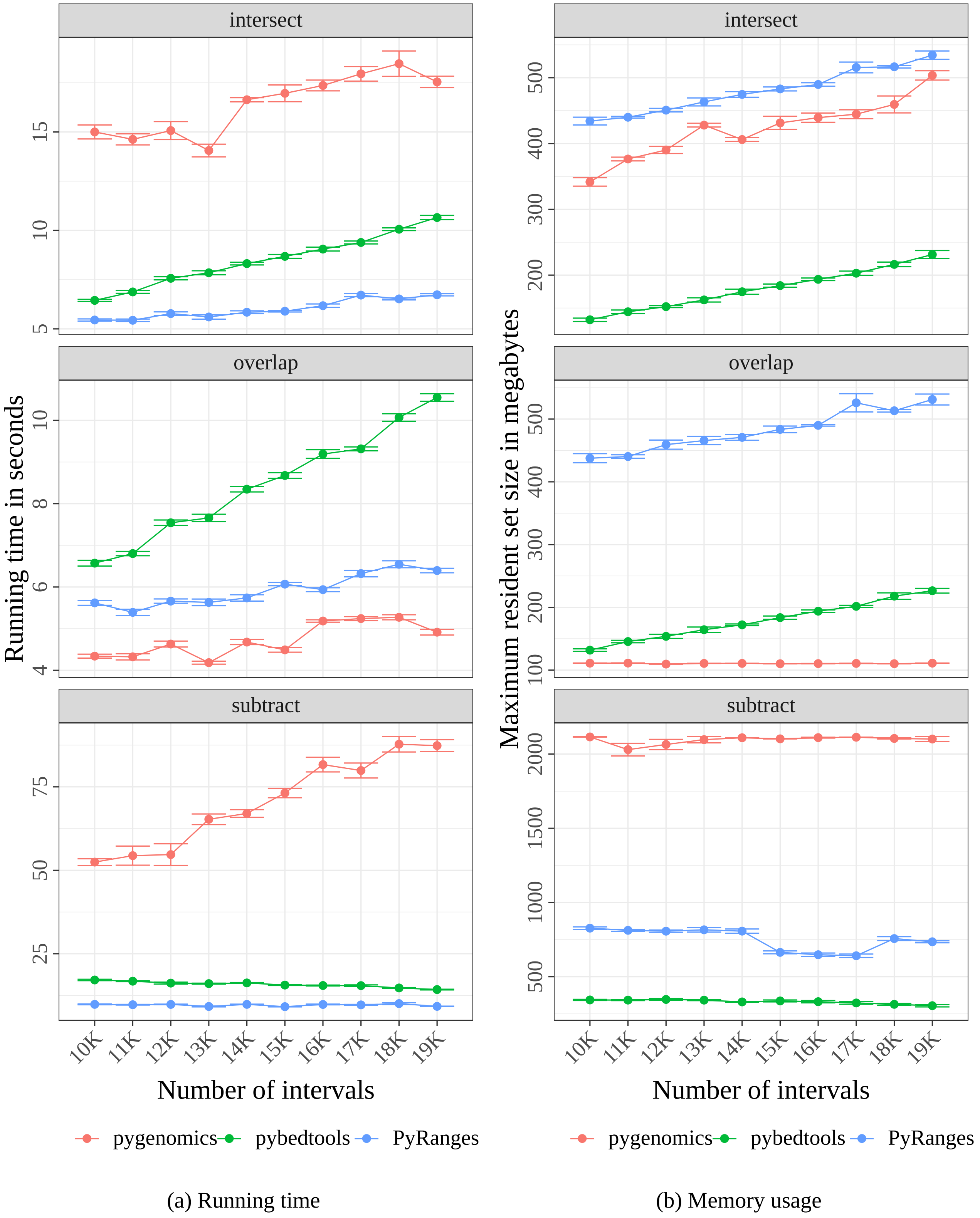

Figure 4: Performance measurements for intersecting, subtracting, and detecting overlapping intervals of protein-coding genes and genomic repeats in the human genome. Points and error bars show two-sided 95% confidence intervals for the mean values.

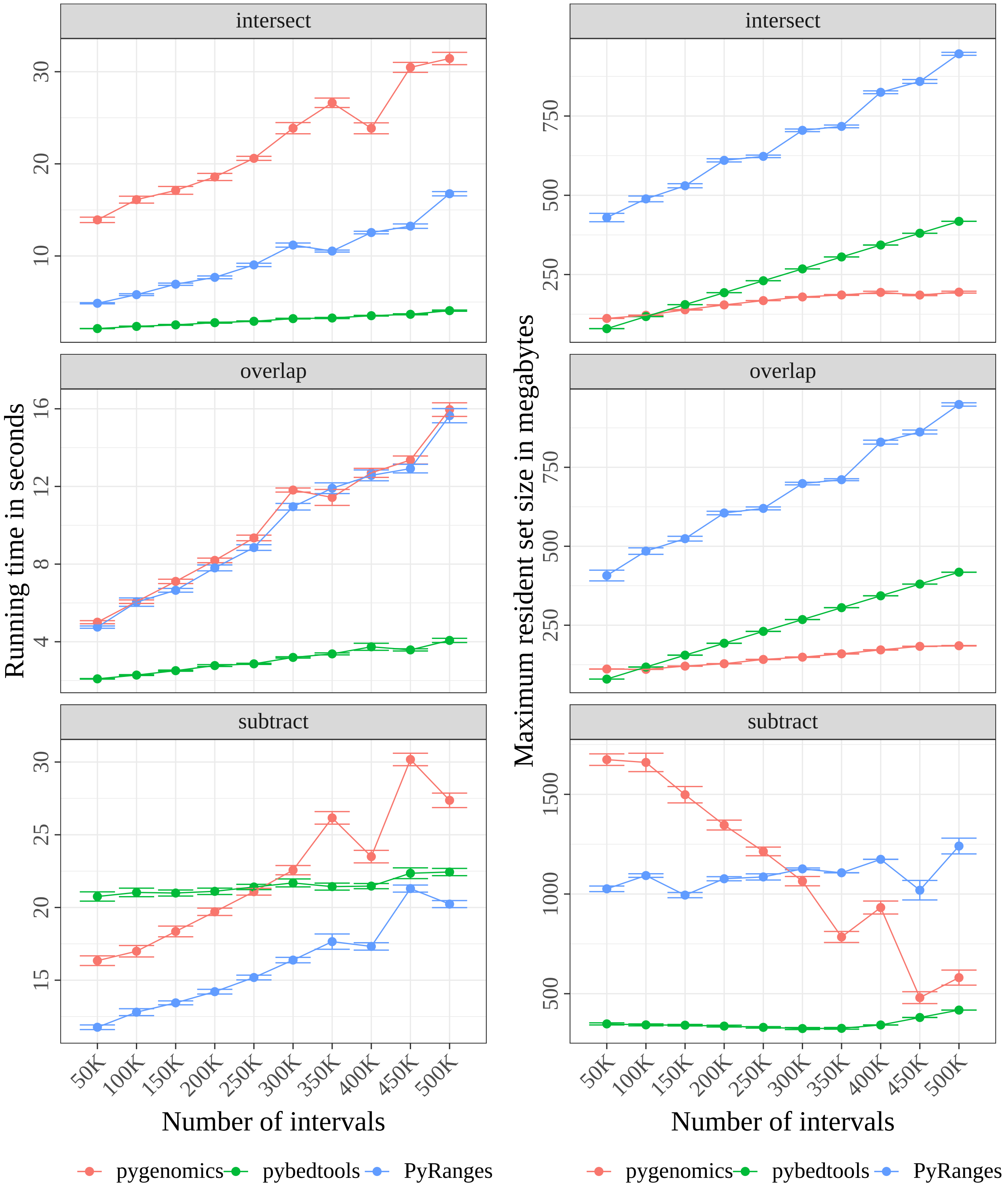

(a) Running time

(b) Memory usage

Figure 5: Performance measurements for intersecting, subtracting, and detecting overlapping intervals of protein-coding gene exons and genomic repeats in the human genome. Points and error bars show two-sided 95% confidence intervals for the mean values.

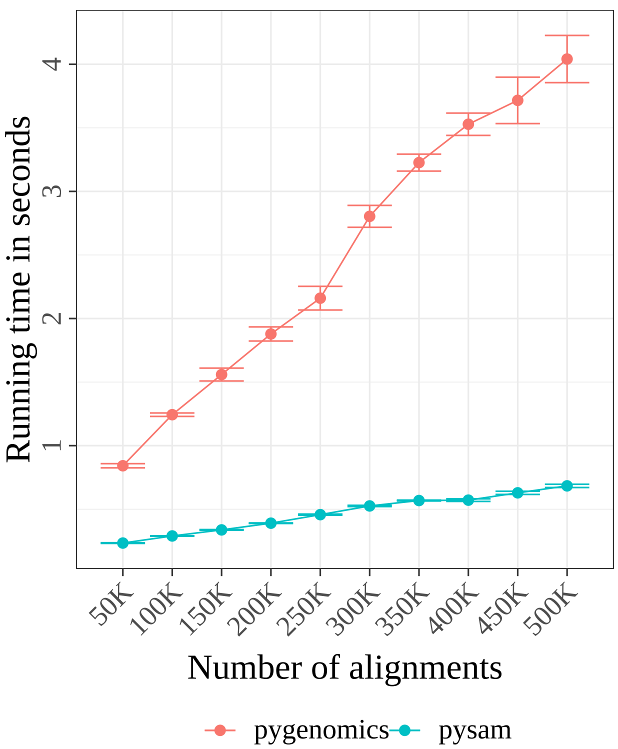

(a) Running time

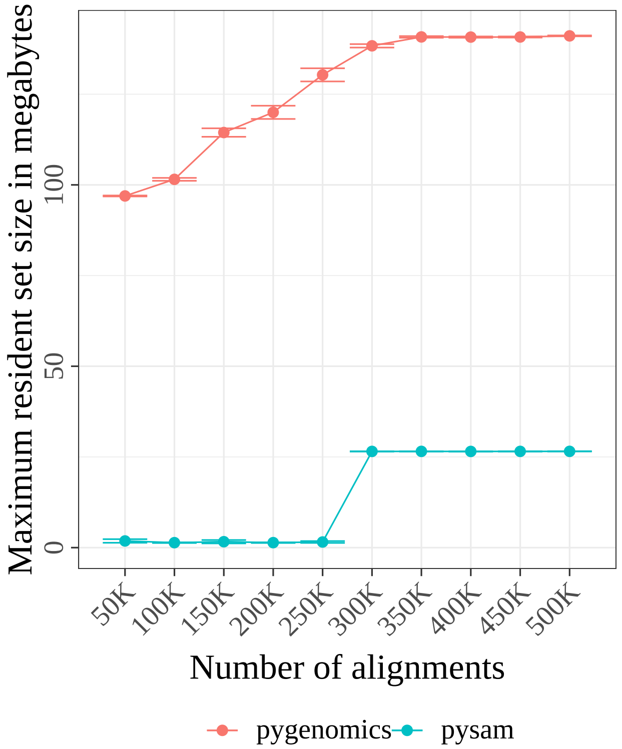

(b) Memory usage

Figure 6: Running time and memory usage for the scripts that iterate read alignments from a BAM file and count properly aligned reads. Points and error bars show two-sided 95% confidence intervals for the mean values.

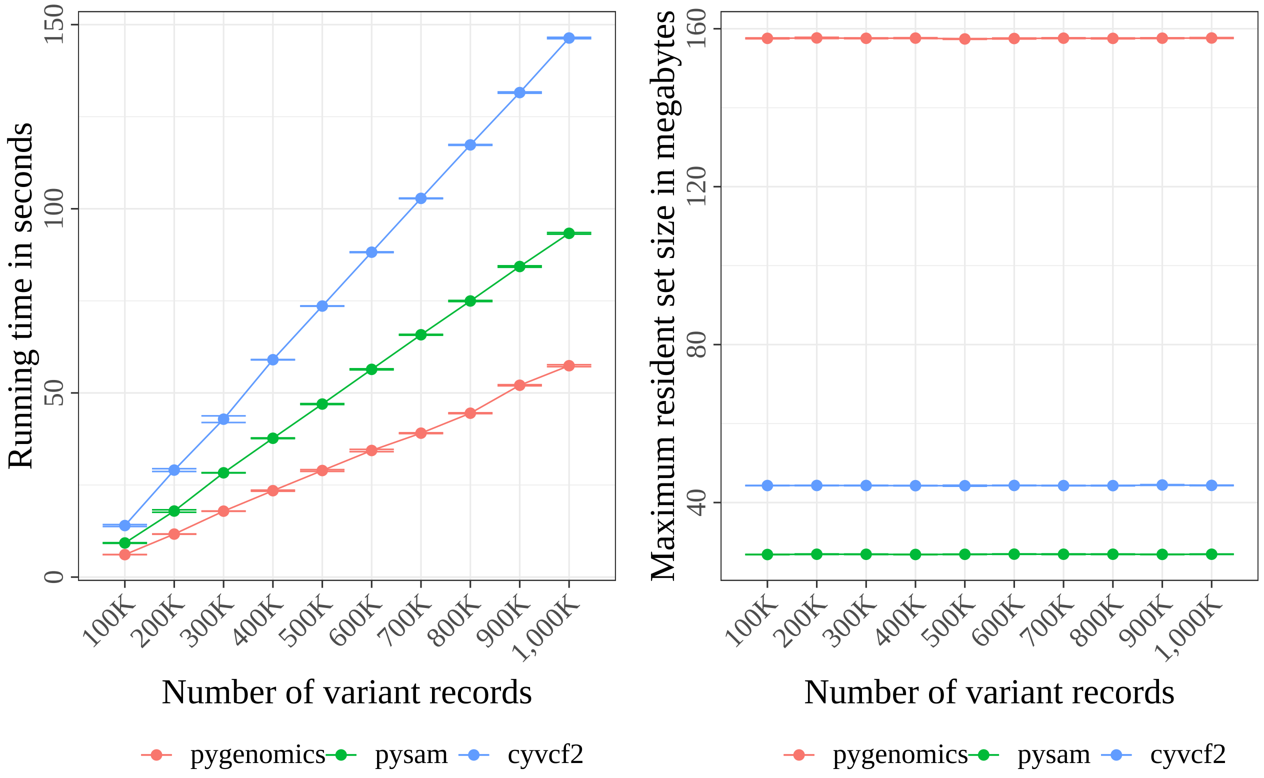

(a) Running time

(b) Memory usage

Figure 7: Running time and memory usage for the scripts that iterate genomic variant records from a VCF file and print biallelic SNPs in the HGVS notation. Points and error bars show two-sided 95% confidence intervals for the mean values.
